# Underage Alcohol Use: Summary of Developmental Processes and Mechanisms: Ages 16–20

**Published:** 2009

**Authors:** Sandra A. Brown, Matthew McGue, Jennifer Maggs, John Schulenberg, Ralph Hingson, Scott Swartzwelder, Christopher Martin, Tammy Chung, Susan F. Tapert, Kenneth Sher, Ken C. Winters, Cherry Lowman, Stacia Murphy

**Keywords:** Adolescence, underage drinking, adolescent drinking, drinking behavior, binge drinking, heavy drinking, alcohol and other drug effects and consequences, risk and protective factors, brain, biological development, cognitive development, emotional development, socialization, decisionmaking

## Abstract

Late adolescence (i.e., the age-group between 16 and 20 years) is characterized by significant changes in neurological and cognitive processes, behavioral and social functioning, and relational and physical contexts as the individual moves toward adulthood. In this age-group, major role transitions affect almost every aspect of life. Moreover, brain development continues—and with it the development of cognitive functions, working memory, emotional and behavioral self-regulation, and decisionmaking. The adolescent’s social and emotional development also continues to evolve, affecting interactions with parents, siblings, peers, and first romantic relationships. All of these changes impact drinking behavior during late adolescence, and, in fact, alcohol use, binge drinking, and heavy drinking are particularly prevalent in youth ages 16–20. Determining the common trajectories of drinking behavior in this age–group is important for understanding how adolescent alcohol use helps shape adult outcomes and for identifying risk and protective factors. It also is important to study the short- and long-term consequences of adolescent alcohol use and abuse, including alcohol’s effects on the developing adolescent brain and accomplishment of important developmental tasks of this age.

The preceding articles in this journal issue have reviewed the developmental processes and mechanisms that are characteristic of childhood and early adolescence. Continuing this review, this article focuses on late adolescence—the period between ages 16 and 20—which is characterized by increasing autonomy and independence as individuals move toward adulthood. (For more information on the definitions of adulthood, see textbox “What Constitutes Adulthood?”) This period is marked by significant changes in neurological and cognitive processes, behavioral and social functioning, and relational and physical contexts. It also is a time when alcohol consumption may escalate and, in many adolescents, can include binge and heavy drinking. In fact, the prevalence of onset of alcohol use disorders (AUDs) is higher in 18- to 20-year-olds than any other time across the life span.

The interrelated cognitive, biological, social, and affective changes that unfold during late adolescence interact and ultimately influence an adolescent’s risk of developing alcohol-related problems. Alcohol involvement in adolescence has short- and long-term consequences on health and well-being. Problematic drinking has the potential to redirect the normative course of adolescent development in ways that increase risk not only for AUDs but also for a range of mental health and social problems. After summarizing the normative development of 16- to 20-year-olds, this article reviews the alcohol use patterns of this age-group, as well as the risk and protective processes shaping alcohol use. Finally, the consequences of adolescent alcohol use and abuse are discussed.

## Normative Development for Ages 16–20: An Overview

During late adolescence, development continues to unfold as new challenges emerge that require adaptation to the increasing responsibilities and changing circumstances of young adulthood. Changes occur both within the individual and in the adolescent’s physical, relational, and social contexts, with major role transitions affecting every domain of life (Schulenberg et al. 1997).

### Developmental Tasks and Transitions

The following are the key developmental tasks and transitions from ages 16 to 20:

- Taking increasing responsibility for one’s daily life, behavior, and future;
- Moving toward less dependent and more mature relationships with the family of origin;
- Moving toward more mature relationships with peers;
- Obtaining a driver’s license and driving, often with a graduated increase in privileges and responsibilities (e.g., learner’s permit, provisional license);
- Exploring romantic and sexual relationships and beginning to date;
- Achieving the age of majority with its associated rights and privileges as well as increased accountability, such as financial responsibility, legal liability, and voting;
- Leaving home and living on one’s own;
- Preparing for and initiating adult occupational roles (e.g., by finishing high school, pursuing postsecondary education, and/or seeking formal paid employment); and
- Cohabitation, engagement, marriage, and/or childbearing for some individuals.

**What Constitutes Adulthood?**

The definition of adulthood depends on the context. Adult legal status is generally determined chronologically, and in the United States and much of the Western world, the “age of majority,” when a person attains most rights, is age 18. The most common rights conferred at this age are voting, contractual capacity, and financial responsibility. Some legal obligations, on the other hand, attach earlier. For example, in most States, adolescents can be held criminally responsible and become subject to the jurisdiction of the criminal court at age 16. Conversely, a few privileges are accorded at a later age, including eligibility to rent a car (minimum age ranging from 21 years up to 25 years) and the right to purchase alcohol (age 21). Developmentalists point to different hallmarks when defining adulthood, including finding a life partner, settling into a career path, and having children. Substantial variability exists in the timing and sequence of these milestones, and today youth are reaching adult independence and responsibilities at later ages than in prior generations. Scientists have termed this extension of adolescence (ages 18–25) “emerging adulthood” (Arnett 2004), which is seen as continued preparation for full adult status.

### Key Developmental Contexts

The period between ages 16 and 20 is characterized by major role transitions in almost every domain of life (Schulenberg et al. 1997). However, although all youth are moving toward greater independence, the timing, sequence, and occurrence of role changes differ greatly among adolescents (Hogan and Astone 1986). Compared with earlier developmental transitions, those that occur during late adolescence are less constrained by age and reflect the greater number of options available to older youth. Therefore, individual differences become increasingly important during late adolescence.

#### Physical Contexts

Although virtually all younger children attend school, for older adolescents this is not the case. For example, 86 percent of adolescents complete high school, but only 62 percent of those enroll in college the year after their high school graduation; others enter the workforce or join the military. Compared with younger adolescents, the living situations of older adolescents are more diverse, reflecting academic and vocational choices as well as family and other circumstances (Brown 2008).

#### Societal and Cultural Influences

The salience of social and cultural influences increases during late adolescence. Their impact on academic achievement, dating and finding a romantic partner, leaving the parental home, becoming a parent, and many other contexts and transitions differs in complex ways by gender, ethnicity, socioeconomic status, neighborhood, and country of origin, in part because of the diversity of available options and supports (Settersten et al. 2005).

## Development Across Multiple Domains

### Brain Maturation

Contrary to what scientists once thought, the brain continues to develop throughout adolescence and into young adulthood, and different regions mature at different times in development (Spear 2000). A number of neuro-maturational processes occur during late adolescence that underlie improvements in cognitive functioning and neural efficiency (Gogtay et al. 2004). These processes include synaptic refinement—which sometimes is referred to as “synaptic pruning” to describe the reduction of synapses and can be detected as a decrease in cortical gray matter volumes (Giedd et al. 1999; Gogtay et al. 2004)—and an increase in myelin,^1^ which can be detected as an increase in white-matter volumes (Giedd et al. 1999; Paus et al. 1999). Together, these changes enhance the efficiency and speed of transmission of neural signals, thereby increasing the capacity for more complex higher-order reasoning and processing. This period of active brain development appears to make the brain more vulnerable to neurotoxic processes, including those attributable to heavy alcohol exposure (Spear 2000).

**Poorly Developed Executive Functions May Predispose to Alcohol Abuse**

Research has confirmed an association between executive functions and alcohol and other drug abuse in adolescents. Poorly developed executive functions are characteristic of youth at high risk for developing alcohol/ substance abuse problems, such as adolescents with conduct disorder, adolescents with high MacAndrew addiction scale scores on the Minnesota Multiphasic Personality Inventory (MMPI), and children of alcoholics. Adolescents with attentional disorders (which by definition indicate impaired executive functioning) have high rates of alcohol and other drug abuse and dependence, especially when they also have a conduct disorder. Executive function scores also predict age at first drink, which is consistent with the finding that students using alcohol prior to the 6th grade have less well-developed decision-making skills. In children of alcoholics, executive function scores predict reactive aggression and the number of drinks per drinking occasion.

In addition, less well-developed executive functioning can combine with coping styles and temperament to increase adolescent vulnerability to substance abuse. Poorer executive functioning in social drinkers has been associated with lessened awareness of abuse consequences and may alter the effectiveness of prevention programs aimed at adolescents.

### Cognitive Development

Executive functions mediate the complex interplay between thinking, emotion, and social judgment. They include goal directedness, independent initiation of behavior, inhibition of inappropriate behavior, flexibility, abstract reasoning, reward appraisal (i.e., evaluating reward likelihood and using reward appraisal to guide behavior), and social appraisal (i.e., understanding social norms and incorporating social information into decisionmaking). Executive functions allow individuals to consider goals, social contexts, and rewards, as well as to regulate response initiation and inhibition. They are pivotal to the two primary cognitive tasks of adolescence: development of the capacity to integrate information from diverse sources relevant to a goal and development of personal rule sets that efficiently guide behavior toward future goals. (For the role of poor executive functions in risk of alcohol abuse, see textbox “Poorly Developed Executive Functions May Predispose to Alcohol Abuse.”)

Part of an adolescent’s improvement in inhibitory control, which develops throughout adolescence, may simply be attributed to increases in the speed of processing within the brain (Christ et al. 2001). The strategic, supervisory, and self-monitoring aspects of executive functioning continue to develop in adolescence, particularly the effective use of available strategies (Suzuki-Slakter 1988). Studies have revealed differences between boys and girls in executive function development. Whereas girls may develop flexible control of verbal processing more quickly than boys, who may not achieve mature verbal control until their late teens (Levin et al. 1991), girls generally lag behind boys in development of the abilities to perform certain nonverbal tasks (Davies 1999).

### Working Memory

The term “working memory” refers to a set of interrelated cognitive processes that result in the ability to temporarily maintain and manipulate information (Baddeley 2000). This ability is central to language comprehension, abstract reasoning, learning, and memory. Verbal and spatial working memory abilities improve throughout childhood and adolescence, and working memory for spatial stimuli in particular continues to mature during adolescence (Luna et al. 2004). For example, older adolescents are more accurate and react more quickly than younger adolescents during tasks involving spatial working memory (Kwon et al. 2002). Brain-imaging research suggests that adolescents and adults activate similar brain regions when performing working memory tasks (Thomas et al. 1999), although adolescents show greater and more widespread activation relative to adults (Klingberg et al. 2002; Kwon et al. 2002). Compared with younger adolescents, older adolescents show increased activation of frontal and parietal brain regions during working memory tasks.

### Development of Emotional and Behavioral Self-Regulation

Self-regulation is the ability to control emotion and behavior, plan behaviors, and resist the impulse to engage in behaviors that result in immediate or delayed negative consequences. Individuals with poorer self-regulation tend to have problems in adolescence and adulthood. One way to measure self-regulation is by maternal report; such studies indicate that self-regulation increases throughout childhood and early adolescence and is relatively stable by late adolescence, with higher levels for girls than boys (Raffaelli et al. 2005). Of note, poor self-regulation early in life, such as the inability or unwillingness to inhibit behavioral impulses in preschool-aged (Caspi et al. 1996) or in elementary school–aged (Elkins et al. 2007) children, predicts AUD risk in late adolescence and early adulthood.

The ability to regulate behavioral impulses is especially important during adolescence, when exposure to risk situations increases. Interestingly, by age 15, adolescents appear to be as capable as adults at logically assessing the likelihood of risk and their own vulnerability to risk; however, adolescents are more likely than adults to engage in risky behaviors (Reyna 2006). Their propensity toward risk taking appears to be linked to the way adolescents process social and emotional cues. Adolescent risk taking may serve a social function because studies show that risk taking increases when adolescents are in the presence of their peers (Gardner and Steinberg 2005). Emotionally charged situations also accelerate adolescent risk taking, possibly reflecting the differential maturation of the brain regions governing emotions compared with those involved in self-regulation (Steinberg 2004).

### Decisionmaking

Decisionmaking is the complex process of choosing an action among different options with various possible outcomes. It involves multiple and sequential use of cognitive processes—for example, logical reasoning (Mueller et al. 2001)—as well as management of reasoning biases and judgment of reasoning success (Klaczynski 2001). All of these abilities develop across adolescence, albeit at different rates (Ormond et al. 1991). In particular, social judgment and social problem solving also improve throughout adolescence into early adulthood (Cauffman and Steinberg 2000).

Decisionmaking is related to the likelihood of alcohol abuse, particularly when decisions are related to risk taking and the assessment of possible consequences. Youth at risk for AUDs and those with more extensive alcohol use histories exhibit a pattern of riskier decisionmaking than nonusing teens (Lejuez et al. 2003). For example, a study involving a gambling task found that although most adolescents performed like adults, adolescents at risk for substance involvement took greater risks in making decisions (Ernst et al. 2003).

### Sleep Changes

In general, a teenager needs approximately 8.5 to 9.25 hours of sleep per night, a requirement that remains stable throughout adolescence. In addition, sleep patterns shift progressively later—that is, teenagers often do not feel physiologically tired until late at night and find it difficult to wake up in the morning. For high-school students who have to get up early, going to bed late results in a sleep deficit because their average weeknight sleep duration is only 7 to 7.75 hours per night (Wolfson and Carskadon 1998). This sleep deficit accumulates over time, despite “catchup” weekend sleep, and results in excessive daytime sleepiness for many. The potential consequences of poor sleep and daytime sleepiness include poor academic performance; accidents from driving while drowsy; negative mood; self-medication with stimulants, alcohol, and other drugs of abuse; and disruption of a variety of cognitive functions (Drummond and Brown 2001; Roehrs et al. 1994).

#### Social and Emotional Development Relationships With Parents

As adolescents move toward adulthood, continued support from, and attachment to, parents remains important (Settersten et al. 2005). The emotional and financial support and sense of security parents can provide may make it easier for adolescents to continue their education and help launch them toward adult life. Although leaving home means that young people spend less time with their parents, the quality of the relationship typically improves (Aseltine and Gore 1993). Importantly, the family values that youth have incorporated generally will continue to inform their choices, including those about alcohol use (Brody et al. 2000). Nevertheless, alcohol involvement increases in parallel with individuation from parents (Baer and Bray 1999) and the decreased parental monitoring that accompanies it (Barnes et al. 2000); moreover, it increases following transition to independent living (Kypri et al. 2004).

#### Siblings

Children from the same family experience influences that are shared (e.g., the environment their parents create in the home) and others which are not (e.g., different friends). In addition, biological siblings have some genes in common. This shared genetic background is one contributor to similar outcomes among siblings. Importantly, siblings also influence one another directly, including with respect to alcohol use. Studies have shown that alcohol use by younger siblings is associated with use by their older brothers and sisters (McGue et al. 1996; Windle 2000). Both the alcohol behavior of older siblings and the younger siblings’ perception of that behavior are important factors. The mechanisms by which older siblings influence substance use may include modeling alcohol use, providing access to alcohol, exerting direct social influence, and influencing cognitions regarding alcohol use.

#### Peer Relationships and Culture

Increases in the importance of peer relationships and exposure to cultural norms and influences may encourage experimentation with alcohol and/or its heavy use (Brown and Abrantes 2005). This experimentation, although potentially dangerous, also may serve a developmental function, particularly with regard to identity exploration and bonding with peers. Certain aspects of social life that are appealing to adolescents may center around drinking contexts, and sociability expressed while drinking may be perceived as an indicator of successful peer relationships and bonding (Maggs 1997). The media further influence adolescents’ perception of use, myths, and other factors that promote experimentation with alcohol. However, the extent of these influences on drinking outcomes varies among adolescents.

Peer influences profoundly affect the risk for alcohol use and abuse in late adolescence (Brown and Abrantes 2005). They may be especially important during periods of change and/or adaptation to new environments. Peers influence adolescents’ drinking behavior through several pathways:

- Modeling and/or directly encouraging specific behaviors, including alcohol use;
- Seeking out and being selected by peers who have similar goals, values, and behaviors;
- Overestimating the prevalence of peer drinking, which can promote heavy drinking; and
- Shifting contexts, such as college or independent living, that alter perceived norms and may minimize the experience of adverse consequences of excessive alcohol use.

The media, which often depict drinking, physical aggression, interpersonal conflict, and unprotected sex, reflect and influence popular culture. Drinking often is portrayed as glamorous and risk-free, thereby augmenting peer influences that encourage alcohol consumption (Brown and Witherspoon 2002).

#### Romantic Relationships and Sexuality

Dramatic changes in sexual feelings, the development of sexual identity, and experimentation with romantic relationships and sexual behaviors are characteristic changes during adolescence. Brooks-Gunn and Paikoff (1997) have identified four developmental challenges in the domain of adolescent sexuality:

- Becoming comfortable with one’s own maturing body;
- Accepting feelings of sexual arousal;
- Understanding that sexual behaviors should be mutually voluntary; and
- Practicing safe sex.

During late adolescence and young adulthood, individuals become intensely interested in finding a romantic partner. Romantic relationships progress from heterosexual group interactions to group dating and, ultimately, to pairing off as couples. During this time, individuals come to rely emotionally more on their romantic partners than their friends (Kuttler and La Greca 2004). Approximately 70 percent of adolescents have engaged in sexual intercourse by the age of 18, most with a single partner and infrequently (Alan Guttmacher Institute 1994).

Early sexual maturation can be a risk for alcohol use among girls. Girls who mature early are more likely to have older boyfriends, which may increase access to alcohol and pressure to drink. Girls whose boyfriends are at least 2 years older become more involved in all forms of sexual intimacy and are more likely to have sex under the influence of alcohol and to experience sexual coercion (Gowen et al. 2004).

Because alcohol often is available at venues frequented by teens, adolescents looking to socialize with peers and/or seeking romantic attachments may be drawn to these contexts. In these situations, positive expectancies about alcohol’s social and sexual enhancement properties can increase the motivation to drink (Goldman 2002). In addition, alcohol lowers inhibitions, which can lead teens to behave in ways they might not otherwise, including unplanned and unprotected sexual activity.

#### Emotional Changes and Mental Health Problems

The neurohormonal changes and brain maturation associated with the onset of and progression through puberty, coupled with the increase in environmental stressors such as school transitions (e.g., progression into high school and college), greater academic demands, transition to first work environments, and exposure to new social situations, contribute to the development of negative emotional states (i.e., negative affect) and emotional volatility and distress. Not surprisingly, numerous mental health problems, including depression, anxiety, suicidal ideation, and delinquent behaviors, increase during this period. These mental health problems often co-occur with AUDs (Abrantes et al. 2003; Kandel et al. 1999). Moreover, intensive and protracted alcohol use can provoke deviant behaviors as well as depression and anxiety (Abrantes et al. 2003; Brown and Abrantes 2005). Youth with comorbid disorders require special attention as they are more difficult to treat successfully (Tomlinson et al. 2004).

## Alcohol Use in the 16–20 Age-Group

By late adolescence, most youth have initiated alcohol use. Between the ages of 16 and 20, drinking, binge drinking, and heavy drinking all increase substantially. As a result, a number of youth begin to experience problems related to their alcohol use.

### Prevalence of Alcohol Use

According to the Monitoring the Future Study, almost two-thirds of 10th-grade students reported having tried alcohol at least once in their lifetime, and two-fifths reported having been drunk at least once (Johnston et al. 2006*a*). Among 12th-grade students, these rates had risen to over three-quarters who reported having tried alcohol at least once and nearly three-fifths who reported having been drunk at least once. In terms of current alcohol use, 33.2 percent of the Nation’s 10th graders and 47.0 percent of 12th graders reported having used alcohol at least once in the past 30 days; 17.6 percent and 30.2 percent, respectively, reported having been drunk in the past 30 days; 21.0 percent and 28.1 percent, respectively, reported having had five or more drinks in a row in the past 2 weeks (sometimes called binge drinking); and 1.3 percent and 3.1 percent, respectively, reported daily alcohol use (Johnston et al. 2006*a*).

Alcohol consumption continues to escalate after high school. In fact, 18-to 24-year-olds have the highest levels of alcohol consumption and alcohol dependence of any age-group. In the first 2 years after high school, lifetime prevalence of alcohol use (based on 2005 follow-up surveys from the Monitoring the Future Study) was 81.8 percent, 30-day use prevalence was 59 percent, and binge-drinking prevalence was 36.3 percent (Johnston et al. 2006*b*). Of note, college students on average drink more than their noncollege peers, even though they drank less during high school than those who did not go on to college (Johnston et al. 2006*a*,*b*; Schulenberg and Maggs 2002). For example, in 2005, the rate of binge drinking for college students (1 to 4 years beyond high school) was 40.1 percent, whereas the rate for their noncollege age mates was 35.1 percent.

Alcohol use and problem drinking in late adolescence vary by sociodemographic characteristics. For example, the prevalence of alcohol use is higher for boys than for girls, higher for White and Hispanic adolescents than for African-American adolescents, and higher for those living in the north and north central United States than for those living in the South and West. Some of these relationships change with early adulthood, however. For example, although alcohol use in high school tends to be higher in areas with lower population density (i.e., rural areas) than in more densely populated areas, this relationship reverses during early adulthood (Johnston et al., 2006 *a*,*b*). Lower economic status (i.e., lower educational level of parents) is associated with more alcohol use during the early high school years; by the end of high school, and during the transition to adulthood, this relationship changes, and youth from higher socioeconomic backgrounds consume greater amounts of alcohol.

### Topography and Trajectories of Drinking

As adolescents seek increasing autonomy, build social and intimate relationships, explore their own identities, and take risks on the way to becoming independent adults, they may believe that alcohol can help them achieve these goals. Therefore, alcohol (and other drug) use during adolescence and early adulthood may be viewed as developmentally driven risk-taking behavior. This underscores the need to consider: (1) the trajectories of alcohol use and problem drinking over time and (2) the relationship of alcohol use and problems to developmental tasks.

#### Trajectories of Alcohol Use and Problem Drinking Over Time

It is essential to investigate how individuals and subgroups of adolescents differ in their patterns of alcohol use (e.g., overall use level, rate of escalation, age at peak use, and rate of decline) as they transition from adolescence into adulthood. This longitudinal approach provides information about both societal trends and common drinking trajectories throughout adolescence.

There are two main types of studies that are used for these purposes. One of these uses an approach called growth models. Findings from such studies indicate that, in general, alcohol use and heavy drinking increase from early adolescence through the early twenties, with marked individual differences in the rates of change. The use of such growth models allows researchers to predict individual differences in levels of use as well as to correlate change in alcohol use with change in other behavioral and social constructs to understand how alcohol use “travels together” with other personal and environmental features.

Another type of study is known as multiple-trajectory study. This approach groups individuals into multiple, relatively homogenous groups according to their distinctive drinking patterns. Analysis of several multiple-trajectory studies (Maggs and Schulenberg 2005) found that the most commonly observed trajectory subgroups in the general population of youth included abstainers; light drinkers; or, very rarely, heavy drinkers across all the time periods measured (see table). Depending on the definitions used in different studies for these levels of alcohol consumption, estimates of the proportion of young people in the low-risk group range from about one-fifth to over two-thirds. In addition, about one-third of adolescents and emerging adults are stable moderate drinkers who engage in some steady but limited heavy drinking across adolescence and young adulthood. Together, these two broad categories comprising relatively low-risk drinkers include a large proportion of all young people.

Many studies also identified groups of chronic heavy drinkers and late-onset heavy drinkers. These two groups are distinguished by the age when the subjects start drinking heavily. Chronic heavy drinkers typically start drinking heavily at a relatively young age and tend not to decrease their drinking in their twenties (e.g., Schulenberg et al. 1996). By contrast, the late-onset heavy-drinking subgroup start to drink later, but their use escalates rapidly. “Fling” drinkers make up 10 percent to 12 percent of the adolescent and young adult population (Schulenberg et al. 1996). They experience a period of developmentally limited heavy drinking that peaks in late adolescence or early adulthood and then declines. Another group, referred to as decreasers, begin heavy drinking at an early age and reduce their use significantly during or shortly after high school. About 10 percent of adolescents and young adults fall into this subgroup that appears to be more common in older adolescent and young adulthood samples than in younger samples. With all of these classifications, however, it is important to keep in mind that substantial fluctuations in adolescent drinking can occur within the course of a single year with 10 to 17 percent of high-school students decreasing or stopping their drinking every year (Brown 2001). These changes may be tied to personal change efforts (Brown 2001) or environmental factors (Greenbaum et al. 2005). Youth receiving alcohol treatment similarly display divergent trajectories (Chung et al. 2003; Myers et al. 2007).

## Risk and Protective Processes

Substantial research has established the major risk and protective factors that influence alcohol use and abuse in late adolescence. For example, adolescent abusers are more likely than nonabusers to:

- Have a positive family history of alcoholism;
- Have preexisting mental health problems;
- Have low levels of self-regulation;
- Have been victims of sexual or physical abuse;
- Come from broken families and/or have parents who poorly monitor their activities;
- Hold beliefs that encourage excessive alcohol use; and
- Be exposed to deviant peer models.

Conversely, adolescents who do not abuse alcohol are more likely to have good relationships with their family, a positive attitude about school, and long-term goals and aspirations.

Studies suggest that both inherited and environmental factors, as well as their interactions, strongly influence drinking behavior (McGue et al. 1996). Importantly, the influence of genes relative to environment varies across drinking behaviors (e.g., initiation, escalation, and dependence); moreover, genetic influences on adolescent drinking behavior appear to strengthen from mid-adolescence to late adolescence (Rose et al. 2001). Finally, whereas some genetic factors that influence problem drinking are specific to alcohol, others influence a range of disinhibited behaviors in late adolescence and early adulthood (Krueger et al. 2002).

Adolescent alcohol use and abuse often co-occur with other adolescent problem behaviors, including tobacco and illicit drug use, early sexual behavior, antisocial behavior, and poor academic performance. It is not clear whether alcohol use increases the likelihood of other adolescent risk behaviors (Kandel et al. 1999) or if underlying factors increase risk for a constellation of problem behaviors (McGue et al. 2001; Vanyukov et al. 2003). Although the two perspectives are different, they are not incompatible. Adolescent alcohol use may reflect an underlying disposition toward under-controlled behavior and also alter the course of adolescent behavior in a way that increases the likelihood of negative outcomes.

In part, the increase in problem drinking during late adolescence may be developmentally driven. Important contextual transitions, such as leaving home/moving out for the first time or moving from high school and entering college, also can significantly influence drinking behavior (Kypri et al. 2004). Studies further suggest that the college environment itself may promote drinking. Whereas college students are less likely to have been binge drinkers in high school than their noncollege peers, they are more likely to binge drink once they enter college (Timberlake et al. 2007). Differences in drinking behavior between college students and their noncollege peers are not necessarily large; however, there may be differences in patterns of drinking (e.g., college students tend to engage in more binge drinking but less daily drinking). The influence of college on drinking behavior is, in most cases, transitory in nature (Bartholow et al. 2003), although it may have long-term consequences. Other transitions also can increase the risk for problem alcohol use if they occur earlier than is typical. For example, teenagers who get married, become parents (Martino et al. 2004), or work more than 20 hours per week (Mortimer and Staff 2004) are at increased risk of problem drinking relative to adolescents whose course of social development is more normative.

During late adolescence, differences in drinking behavior between boys and girls begin to emerge and become consequential (Johnston et al. 2006*a*). For example, adolescent girls are as likely as adolescent boys to have tried alcohol but are less likely to engage in problematic or binge drinking or be diagnosed with an AUD. The factors that put boys and girls at risk also can be different. For example, adolescent girls are more likely than adolescent boys to be victims of abuse (Champion et al. 2004) and to suffer from depression and anxiety (Poulin et al. 2005), both of which are associated with elevated rates of drinking. Adolescent boys, in contrast, are more likely to suffer from externalizing psychopathology and to score high on measures of impulsivity, which are associated with elevated drinking rates (Caspi et al. 1996). Importantly, adolescents who exhibit a range of developmental psychopathology (both internalizing and externalizing) are among those at highest risk for excessive alcohol involvement.

### Resilience

The term “resilience” denotes positive developmental outcomes in individuals despite significant adversity in their lives. This can mean either that potential problems fail to emerge or that existing problems resolve under adverse circumstances. Patterns of resilient behavior can provide important information about normal development as well as the development of problem behaviors and may provide insight into effective preventive strategies. The concept of resilience is important in understanding how youth with genetic or environmental risk factors for alcohol dependence can overcome their developmental liabilities (Zucker et al. 2003). Biological, cognitive, social, and emotional characteristics all can contribute to resilience. In particular, certain integrative skills, such as self-organization and emotional regulation that emerge in late adolescence, promote resilience and may protect against trajectories of protracted heavy alcohol involvement.

## The Consequences of Adolescent Alcohol Use and Abuse

Another important aspect of studies of underage drinking is to determine how alcohol consumption during adolescence affects subsequent development. Some of the challenges are to understand to what extent outcomes are directly related to alcohol use and to evaluate the relative influence of factors that precede or occur with alcohol involvement. For example, early externalizing disorders (e.g., conduct disorder) are associated with risk for a range of enduring, adverse behavioral outcomes, including excessive alcohol and other drug involvement. Adolescent alcohol involvement itself can disrupt social and emotional development as well as interfere with academic progress.

More specifically, alcohol-related problems and consequences include social problems (e.g., physical or verbal aggression and relationship difficulties), legal problems (e.g., arrests for driving while intoxicated and public inebriation), educational/vocational problems (e.g., academic difficulties, termination from employment, and failure to achieve career goals), and medical problems (e.g., unintentional injury, liver disease, and central nervous system disease). A variety of studies have demonstrated associations between adolescent alcohol involvement and a range of adverse consequences, including academic problems, social problems, hangovers, unplanned and risky sex, aggression and victimization, unintentional injuries (especially motor vehicle crashes), various physical and emotional problems, and suicidality (Tomlinson et al. 2004). These consequences may directly result from alcohol consumption and/or from factors associated with drinking (Stice et al. 1998). For certain outcomes, their relationship with alcohol consumption is clear (e.g., experiencing blackouts or alcohol-related arrests or having other people complain about one’s drinking). For other outcomes, the relationship may be less apparent (e.g., getting into fights, skipping a class because of one’s drinking, or suffering from depression). Therefore, adolescents may not attribute these problems to their alcohol consumption even if they are a consequence of alcohol involvement (e.g., conflict with parents).

### Alcohol and the Developing Adolescent Brain

During adolescence, the brain undergoes significant maturation in specific regions. As noted in the preceding article by Windle and colleagues (see pp. 30–40 of this issue), the limbic system, which regulates emotional tone and reactivity, matures in early adolescence. In contrast, self-regulation and a broad range of higher-level cognitive functions, which are governed by the frontal cortex, develop more gradually throughout adolescence and into early adulthood as a function of age and experience.

Late adolescence is a time when many individuals escalate their drinking, raising concerns about effects of heavy drinking on neurodevelopment during this period. Research on adolescent animals as well as limited studies with human adolescents show the potential for adolescent alcohol use to induce functional and/or structural changes in the brain as well as neurocognitive deficits (McQueeny et al. 2009; Spear 2000). For example, in a study of adolescents with AUDs, protracted heavy alcohol use was associated with deficits in memory retrieval and visuospatial functioning (Brown et al. 2000). In addition, imaging studies of brain structure (Nagel et al. 2004) and longitudinal neurocognitive evaluations of clinical and community samples of adolescent drinkers (Brown et al. 2000) found differences between heavily alcohol-involved adolescents and control subjects. Thus, late adolescents and young adults who persisted in heavy drinking showed greater deficits; however, it is not yet clear what levels and duration of alcohol exposure produce significant and long-lasting changes in brain function and what other factors may contribute to these changes.

Research has shown that adolescent animals are more vulnerable than adults to the adverse effects of alcohol on specific brain regions, including the hippocampus and areas of the frontal cortex (Spear 2000). For example, acute alcohol exposure in rats at both high and low doses inhibits memory formation, which is a function of the hippocampus, with adolescent rats being especially sensitive to these effects. Animal research also has shown that repeated exposure to very high levels of alcohol is particularly harmful to the brain and is associated with reduced formation of new nerve cells (i.e., neurogenesis) in the rat hippocampus (Nixon and Crews 2002) and long-term alteration of brain-signaling functions involving the neurotransmitter serotonin (Obernier et al. 2002). Taken together, these lines of research heighten concern about the patterns of binge drinking (so prevalent among human adolescents) that can result in extremely high blood alcohol levels.

Animal studies also suggest that, in contrast to their elevated sensitivity to the cognition-impairing effects of alcohol, adolescents are less sensitive than adults to other effects of alcohol. For example, adolescent rats are less sensitive to alcohol’s motor-impairing and sedating effects (Little et al. 1996). If human adolescents also are less sensitive to these cues, which typically moderate alcohol consumption (i.e., sedation and locomotor impairment), this may explain, at least in part, their typical patterns of drinking. Further, it appears that in adolescent, but not adult, rats repeated exposure to very high levels of alcohol may further reduce sensitivity to these aversive effects (Graham and Diaz-Granados 2006) and has been associated with increased tolerance. In humans, reduced sensitivity to the motor-impairing and aversive effects of alcohol has been shown to be a potent risk factor for the development of alcoholism (Schuckit and Smith 1996). Therefore, the reduced sensitivity that can result from heavy alcohol use during adolescence may contribute to increased risk for future alcohol dependence.

### Adolescent Alcohol Involvement and Future Alcohol and Other Substance Involvement

Several studies have followed adolescent drinkers into their third decade of life. In general, these studies found that drinking patterns during late adolescence are associated with future negative alcohol-related outcomes, including diagnoses of AUDs during the third decade of life (Chassin et al. 2002; Wells et al. 2004). In addition, studies found that greater intensity of use (e.g., greater maximum number of drinks per occasion in the recent past at age 16) predicted a higher likelihood of being diagnosed with an AUD (Wells et al. 2004). Thus, the relationship between alcohol involvement in later adolescence and alcohol involvement in early adulthood appears to be robust across a number of studies, even when controlling for a variety of competing factors.

Additional studies have attempted to relate various aspects of middle- or late-adolescent drinking behavior to later outcomes in developmentally important domains other than alcohol use. Although strong associations exist between drinking patterns at age 16 and educational, occupational, and emotional outcomes, few can be attributed exclusively to early alcohol use. When demographic and background variables are controlled, alcohol involvement at age 16 appears to independently predict noncannabis drug dependence at age 25, number of sexual partners, and property and violent offenses (Wells et al. 2004).

Longitudinal studies that investigated the long-term correlates of late-adolescent drinking found that alcohol involvement in adolescence was associated with adult drug dependence, antisocial behavior, and depression (Hill et al. 2000; Wells et al. 2004). Adult antisocial behavior is associated with being drunk by age 18 even in the absence of childhood conduct problems. Less is known about the association between adolescent alcohol involvement and other domains of adult functioning. For example, although adolescent alcohol involvement is associated with educational problems in both secondary and higher education, it is not clear if the effects are uniquely attributable to alcohol.

## Conclusion

Late adolescence spans ages 16–20. It is a period of extensive and rapid transition in virtually every domain of life functioning, as well as of continued neurologic, cognitive, and social maturation. Increased autonomy, reduced parental monitoring, and greater involvement with peers all create the opportunities for psychological growth as well as a context for the emergence of problem drinking. In fact, during late adolescence many youth escalate their drinking, resulting for some in alcohol-related problems that may include AUDs. Although problem drinking may represent a transient phase in the lives of many adolescents, for others it can have profound and life-altering effects.

This article has reviewed some of the major developmental processes, transitions, and tasks in late adolescence as they relate to alcohol use and its consequences, including developmentally related effects and alcohol-specific risk and protective factors. The following article by Spoth and colleagues (pp. 53–66) examines the prevention and reduction of alcohol use and AUDs in adolescents.

## Figures and Tables

**Table:** Alcohol Use Trajectory Groups Identified in Community-Based Research on Adolescents

**Trajectory Group**	**Alcohol Use Pattern**	**Approximate Representation (%)**
Abstainers/light drinkers	Stable low use or nonuse of alcohol	∼20–65
Stable moderate drinkers	Stable moderate use, limited heavy use	∼30
Fling drinkers	Time-limited periods of heavy use	∼10
Decreasers	Early onset but declining course of alcohol use	∼10
Chronic heavy drinkers	Early onset and stable course of heavy drinking	<10
Late-onset heavy drinkers	Late onset with rapid escalation to heavy drinking	<10

SOURCE: Brown et al. 2008.

## Footnotes

1 Myelin is a white fatty material composed mainly of alternating layers of lipids and lipoproteins that covers the extensions of many nerve cells in order to insulate them and make nerve signal transmission faster and more effective. Because of myelin’s white color, myelin-covered nerve fiber bundles in the brain are referred to as white matter, as opposed to gray matter, which refers to brain structures composed mainly of nerve cell bodies.
